# An overview of DNA methylation markers for early detection of gastric cancer: current status, challenges, and prospects

**DOI:** 10.3389/fgene.2023.1234645

**Published:** 2023-07-25

**Authors:** Ying Xue, Chao Huang, Bing Pei, ZhenZhen Wang, Yanmiao Dai

**Affiliations:** ^1^ The Affiliated Suzhou Hospital of Nanjing Medical University, Suzhou Municipal Hospital, Gusu School, Nanjing Medical University, Suzhou, China; ^2^ Department of Clinical Laboratory, The Affiliated Suqian First People’s Hospital of Nanjing Medical University, Suqian, Jiangsu, China; ^3^ Department of Laboratory Medicine, Affiliated Xuzhou Maternity and Child Healthcare Hospital of Xuzhou Medical University, Xuzhou, China; ^4^ Department of Spleen and Stomach Diseases, Kunshan Hospital of Traditional Chinese Medicine, Kunshan, China

**Keywords:** sample types, analytical methods, non-invasive, gastric cancer, DNA methylation

## Abstract

**Background:** Gastric cancer (GC) is one of the most common malignancies, with a low 5-year survival rate. However, if diagnosed at an early stage, it can be cured by endoscopic treatment and has a good prognosis. While gastrointestinal X-ray and upper endoscopy are used as national GC screening methods in some GC high-risk countries, such as Japan and Korea, their radiation exposure, invasiveness, and high cost suggest that they are not the optimal tools for early detection of GC in many countries. Therefore, a cost-effective, and highly accurate method for GC early detection is urgently needed in clinical settings. DNA methylation plays a key role in cancer progression and metastasis and has been demonstrated as a promising marker for cancer early detection.

**Aims and methods:** This review provides a comprehensive overview of the current status of DNA methylation markers associated with GC, the assays developed for GC early detection, challenges in methylation marker discovery and application, and the future prospects of utilizing methylation markers for early detection of GC. Through our analysis, we found that the currently reported DNA methylation markers related to GC are mainly in the early discovery stage. Most of them have only been evaluated in tissue samples. The majority of non-invasive assays developed based on blood lack standardized sampling protocols, pre-analytical procedures, and multicenter validation, and they exhibit insufficient sensitivity for early-stage GC detection. Meanwhile, the reported GC DNA methylation markers are generally considered pan-cancer markers.

**Conclusion:** Therefore, future endeavors should focus on identifying additional methylation markers specific to GC and establishing non-invasive diagnostic assays that rely on these markers. These assays should undergo multicenter, large-scale prospective validation in diverse populations.

## 1 Introduction

Gastric cancer (GC) is a major global health concern, it caused 1,089,103 new cases and 768,793 new deaths in 2020, ranking fifth for incidence and fourth for mortality among all cancer types globally (Sung et al., 2021). However, more than 60.0% GC new cases were found in Eastern Asian countries, such as China, Japan, Korea and Mongolia (Ning et al., 2022; Sekiguchi et al., 2022). Although the GC incidence was remarkable decreasing during the past several decades with the improvement of medical treatments and public health strategies (Sekiguchi et al., 2022; Song et al., 2022), the 5-year survival rate for GC still relatively low (about 30.0%–40.0%) in most countries (Sekiguchi et al., 2022). In contrast, 2 GC high-risk countries, Japan and Korea, reported a high 5-year survival rate for GC of 60.0%–70.0%, which was due to the long-term national GC screening programs, and many of these cases were found at early stage (Hamashima, 2020; Sekiguchi et al., 2022). In Japan and Korea, the GC screening strategies included gastrointestinal X-ray and upper endoscopy detection. However, because upper endoscopy is an invasive method with several side effects and a low compliance rate, and gastrointestinal X-ray suffer the risk of radiation exposure, they were not the best choice for primary screening of GC. Other non-invasive methods, such as the CA72-4, PGI/II and the ABC method (combination assay of *Helicobacter pylori* and serum pepsinogen), have insufficient sensitivity and specificity (Yamaguchi et al., 2016; Xu Y. et al., 2021).

DNA methylation is the most widely studied epigenetic modification which plays a significant role in cancer progression and metastasis (Davalos and Esteller, 2022). Many of DNA methylation occur early in tumorigenesis, which allows DNA methylation-based markers to be suitable for early detection of cancer (Locke et al., 2019; Zhao et al., 2019; Zhao et al., 2020). Because DNA methylation can also be detected in body fluids such as blood, stool, urine, and cerebrospinal fluid (Liu et al., 2020; Rahat et al., 2020), it is more stable, sensitive and specific than other cell free nucleic acid markers (miRNA, lncRNA or mRNA), indicating that DNA methylation is a promising non-invasive marker for cancer early detection (Jamshidi et al., 2022). During the past decade, several commercially available DNA methylation‐based assays have been developed and approved by the United States Food and Drug Administration (FDA) and Chinese National Medical Products Administration (NMPAfor clinical application. For example, the plasma *SPET9* methylation test (Epi proColon) (Church et al., 2014), and the stool multiple-target assay (Cologuard), which contained two methylation markers (Imperiale et al., 2014), were successfully used for colorectal cancer (CRC) screening (Wolf et al., 2018). Moreover, many studies have proven that DNA methylation markers are more sensitive and specific than traditional blood protein markers (Young et al., 2016; Cai et al., 2021; Lin et al., 2021). Promoter CpG island hypermethylation and tumor suppressor genes inactivated in gastric carcinogenesis has also been frequently observed (Patel et al., 2017), thus DNA methylation can also be a potential marker for GC early detection. Recent advances in translational genomics and analytics, drives numerous potential DNA methylation markers have come to light in clinics.

This review provides a comprehensive overview of the DNA methylation changes in GC, and summarized the achievements, challenges and possible further direction for DNA methylation as a potential tool for GC early detection.

## 2 Sample types and analytical methods for DNA methylation analysis

In this review, we performed an extensive search and analysis of previously published DNA methylation markers to evaluate their effectiveness in the detection of GC across different sample types (Table 1; Table 2; Table 3). According to our analysis, current research on DNA methylation in GC primarily focuses on tissue, gastric washes/juice, blood (plasma or serum) and stool samples. Tissue samples are primarily employed for the initial identification of methylation markers, whereas blood or stool samples are commonly used for the subsequent validation of identified markers. Regarding the sample types used in the previous studies, we found that plasma was the most commonly used sample type for DNA methylation analysis, with 26 studies utilizing this sample type. However, in 6 of these studies, plasma volumes were ≤1mL, which may have impacted the accuracy and sensitivity of the analysis. In contrast, 3.5 mL of plasma was used in 7 studies. Serum was used in 13 studies, and the volume of serum used was ≤0.5 mL in 53.8% of these studies.

**TABLE 1:** DNA methylation markers individually evaluated for GC detection.

Markers	Authors	Year	Country	Sample type	Number of subjects	Method	SN (%)	Stage I SN (%)	SP (%)	AUC
P16	Lee et al. (2002)	2002	China (Hong Kong)	Tissue	44 GC	MSP	66.7	71.4	—	—
				Serum	44 GC, 30 Control	MSP	51.9	57.1	100.0	—
P16	Kanyama et al. (2003)	2003	Japan	Tissue	60 GC, 60 Control	MSP	38.3	—	100.0	—
				Serum	60 GC, 16 Control	MSP	10.0	—	100.0	—
P16	Koike et al. (2004)	2004	Japan	Serum	41 GC, 10 Control	MSP	22.0	—	100.0	—
P16	Ichikawa et al. (2004)	2004	Japan	0.4 mL serum	109 GC, 10 Control	MSP	18.3	—	100.0	—
P16	Hou et al. (2005)	2005	China	Tissue	60 GC, 60 Control	Nested-MSP	86.7	—	100.0	—
P16	Abbaszadegan et al. (2008)	2008	Iran	Tissue	52 GC, 50 Control	MSP	44.2	—	100.0	—
				Serum	52 GC, 50 Control	MSP	26.9	—	100.0	—
P16	Hu et al. (2010)	2010	China	Tissue	70 GC, 30 Control	MSP	68.6	—	100.0	—
P16	Saliminejad et al. (2020)	2020	Iran	2 mL plasma	96 GC, 88 Control	MSP	41.7	—	84.1	0.63
RNF180	Cheung et al. (2012)	2012	China (Hong Kong)	Tissue	198 GC, 20 IM, 23 Control	Sanger Sequencing	GC: 75.8, IM: 55.0	—	100.0	—
				0.8 mL plasma	109 GC, 190 Control	qMSP	56.3	—	100.0	—
RNF180	Zhang et al. (2014)	2014	China	0.4 mL plasma	57 GC, 42 Control	MSP	57.9	—	76.2	—
RNF180	Cao et al. (2020)	2020	China	3.5 mL plasma	74 GC, 99 BGD, 57 NED	qMSP	GC: 32.4, GD: 13.1	—	86.9	0.64
RNF180	Xu et al. (2021b)	2021	China	3.5 mL plasma	151 GC, 56 AG, 87 Other GIDs, 224 NED	qMSP	GC: 37.1, AG: 19.8, Other GIDs: 14.3	<20	88.4	0.72
RNF180	Zhao et al. (2022a)	2022	China	3.5 mL plasma	60 GC, 96 Control	qMSP	71.7	—	59.4	0.73
RUNX3	Sakakura et al. (2009)	2009	Japan	0.4 mL serum	65 GC, 50 Control	qMSP	29.2	—	100.0	—
RUNX3	Hu et al. (2010)	2010	China	Tissue	70 GC, 30 Control	MSP	60.0	—	100.0	—
RUNX3	Lin et al. (2017)	2017	China	0.4 mL plasma	131 GC, 56 IN, 30 IM, 34 Control	MSP	GC: 42.7	—	79.2	—
RUNX3	Saliminejad et al. (2020)	2020	Iran	2 mL plasma	96 GC, 88 Control	MSP	58.3	—	95.5	0.77
RUNX3	Hideura et al. (2020)	2020	Japan	0.4 mL serum	50 GC, 61 Control	CORD Assay	50.0	—	80.3	0.70
SEPT9	Cao et al. (2020)	2020	China	3.5 mL plasma	74 GC, 99 BGD, 57 NED	qMSP	GC: 28.4, BGD: 6.1	—	94.7	0.62
SEPT9	Xu et al. (2021b)	2021	China	3.5 mL plasma	151 GC, 56 AG, 87 Other GIDs, 224 NED	qMSP	GC: 48.3, AG: 9.3, Other GIDs: 6.7	<15	98.2	0.77
SEPT9	Zhao et al. (2022b)	2022	China	3.5 mL plasma	60 GC, 96 Control	qMSP	48.3	—	86.9	0.70
Reprimo	Bernal et al. (2008)	2008	Chile	Plasma	43 GC, 31 Control	MSP	95.3	—	90.3	—
Reprimo	Liu and Yang (2015)	2015	China	Plasma	50 IM, 50 Dysplasia, 50 GC, 30 Control	MSP	IM: 28.0, Dysplasia: 56.0, GC: 62.0	—	100.0	—
Reprimo	Wang et al. (2016)	2016	China	Tissue	42 GC, 28 Control	MS-MCA	70.0	—	46.4	—
				1 mL Serum	35 GC, 28 Control	MS-MCA	94.3	—	92.9	—
E-cadherin	Lee et al. (2002)	2002	China (Hong Kong)	Tissue	44 GC	MSP	75.9	71.4	—	—
				Serum	44 GC, 30 Control	MSP	57.4	57.1	100.0	—
E-cadherin	Ichikawa et al. (2004)	2004	Japan	0.4 mL serum	109 GC, 10 Control	MSP	23.8	—	100.0	—
E-cadherin	Koike et al. (2004)	2004	Japan	Serum	41 GC, 10 Control	MSP	22.0	—	100.0	—
ZIC1	Chen et al. (2015)	2015	China	Plasma	104GC, 50 IN, 20 Control	MSP	GC: 60.6, IN: 54.0	—	100.0	—
ZIC1	Lin et al. (2017)	2017	China	0.4 mL plasma	131 GC, 56 IN, 30 IM, 34 Control	MSP	GC: 69.5	—	69.2	—
ELMO1	Maeda et al. (2018)	2018	Japan	Tissue	52 GC, 50 Control	Pyrosequencing	—	—	—	0.75
ELMO1	Peng et al. (2022)	2022	China	3.5 mL plasma	32 GC, 64 Control	qMSP	33.9	—	100.0	0.64
TFPI2	Hibi et al. (2011a)	2011	Japan	0.2 mL serum	73 GC, 20 Control	qMSP	9.6	—	100.0	—
TFPI2	Peng et al. (2022)	2022	China	3.5 mL plasma	109 GC, 190 Control	qMSP	27.5	—	97.9	0.63
RASSF1A	Pimson et al. (2016)	2016	Thailand	0.2 mL plasma	101 GC, 202 Control	MSP	83.2	—	94.6	—
RASSF1A	Saliminejad et al. (2020)	2020	Iran	2 mL plasma	96 GC, 88 Control	MSP	33.3	—	100.0	0.67
SFRP2	Zhang et al. (2014)	2014	China	0.4 mL plasma	57 GC, 42 Control	MSP	71.9	—	57.1	—
SFRP2	Miao et al. (2020)	2020	China	3.5 mL plasma	92 GC, 16 IM, 26 GFGP, 13 AP, 39 HP, 50 Control	qMSP	GC: 60.9, IM: 56.3, GFGP: 34.6, AP: 23.1, HP: 30.8	50.0	86.0	0.78
RPRM	Maeda et al. (2018)	2018	Japan	Tissue	52 GC, 50 Control	Pyrosequencing	—	—	—	0.75
RPRM	Saliminejad et al. (2020)	2020	Iran	2 mL plasma	96 GC, 88 Control	MSP	66.7	—	93.2	0.80
DAPK	Lee et al., 2002)	2002	China (Hong Kong)	Tissue	44 GC	MSP	70.3	57.1	—	—
				Serum	44 GC, 30 Control	MSP	48.1	28.6	100.0	—
DAPK	Hu et al. (2010)	2010	China	Tissue	70 GC, 30 Control	MSP	60.0	—	100.0	—
GSTP1	Lee et al. (2002)	2002	China (Hong Kong)	Tissue	44 GC	MSP	18.5	14.3	—	—
				Serum	44 GC, 30 Control	MSP	14.8	14.3	100.0	—
p15	Lee et al. (2002)	2002	China (Hong Kong)	Tissue	44 GC	MSP	68.5	71.4	—	—
				Serum	44 GC, 30 Control	MSP	55.6	42.9	100.0	—
RARb	Koike et al. (2004)	2004	Japan	Serum	41 GC, 10 Control	MSP	14.6	—	100.0	—
NMDAR2B	Liu et al. (2007)	2007	China	Tissue	28 GC, 20 Control	qMSP	60.7	—	95.0	—
CDH1	Muretto et al. (2008)	2008	Italy	Gastric juice	20 GC, 14 Control	MSP	65.0	—	100.0	—
MINT25	Watanabe et al. (2009)	2009	United States, Japan and Korea	Tissue	22 Control, 40 Dysplasias, 91 GC	Pyrosequencing	84.1	—	90.9	0.94
				Gastric washes	20 GC, 48 Control	Pyrosequencing	90.0	—	95.8	0.96
RORA	Watanabe et al. (2009)	2009	United States, Japan and Korea	Tissue	22 Control, 40 Dysplasias, 91 GC	Pyrosequencing	83.2	—	86.4	0.89
				Gastric washes	20 GC, 48 Control	Pyrosequencing	60.0	—	85.4	0.71
PRDM5	Watanabe et al. (2009)	2009	United States, Japan and Korea	Tissue	22 Control, 40 Dysplasias, 91 GC	Pyrosequencing	64.2	—	94.7	0.75
				Gastric washes	20 GC, 48 Control	Pyrosequencing	65.0	—	93.7	0.83
MLF1	Watanabe et al. (2009)	2009	United States, Japan and Korea	Tissue	22 Control, 40 Dysplasias, 91 GC	Pyrosequencing	61.8	—	81.8	0.73
				Gastric washes	20 GC, 48 Control	Pyrosequencing	60.0	—	85.4	0.68
ADAM2	Watanabe et al. (2009)	2009	United States, Japan and Korea	Tissue	22 Control, 40 Dysplasias, 91 GC	Pyrosequencing	65.9	—	90.0	0.81
				Gastric washes	20 GC, 48 Control	Pyrosequencing	70.0	—	83.3	0.86
GDNF	Watanabe et al. (2009)	2009	United States, Japan and Korea	Tissue	22 Control, 40 Dysplasias, 91 GC	Pyrosequencing	81.9	—	90.9	0.88
				Gastric washes	20 GC, 48 Control	Pyrosequencing	65.0	—	89.6	0.74
CHFR	Hu et al. (2010)	2010	China	Tissue	70 GC, 30 Control	MSP	48.6	—	100.0	—
RECK	Du et al. (2010)	2010	China	Tissue	40 GC, 40 Control	MSP	47.5	—	76.5	—
CHRM2	Chen et al. (2012)	2012	China	Serum	58 GC, 46 GPL, 30 Control	MSP	GC: 31.1, GPL: 15.2	—	93.3	—
FAM5C	Chen et al. (2012)	2012	China	Serum	58 GC, 46 GPL, 30 Control	MSP	GC: 31.0, GPL: 6.5	—	96.7	—
MYLK	Chen et al. (2012)	2012	China	Serum	58 GC, 46 GPL, 30 Control	MSP	GC: 70.7, GPL: 28.3	—	93.3	—
Sox17	Oishi et al. (2012)	2012	Japan	Gastric washes	64 GC, 64 Control	Pyrosequencing	—	20.6	—	—
VIM	Shirahata et al. (2012)	2012	Japan	0.2 mL serum	74 GC	qMSP	33.8	41.2	—	—
					21 Control					
BCL6B	Yang et al. (2013)	2013	China (Hong Kong)	1 mL plasma	40 GC, 22 Control	Sanger sequencing	42.5	—	100.0	—
DAPK1	Zhang et al. (2014)	2014	China	0.4 mL plasma	57 GC, 42 Control	MSP	49.1	—	71.4	—
CDO1	Vedeld et al. (2015)	2015	Norway	FFPE	25 GC	qMSP	88.0	—	—	—
DCLK1	Vedeld et al. (2015)	2015	Norway	FFPE	25 GC	qMSP	96.0	—	—	—
SFRP1	Vedeld et al. (2015)	2015	Norway	FFPE	25 GC	qMSP	92.0	—	—	—
ZNF331	Vedeld et al. (2015)	2015	Norway	FFPE	25 GC	qMSP	80.0	—	—	—
ZSCAN18	Vedeld et al. (2015)	2015	Norway	FFPE	25 GC	qMSP	76.0	—	—	—
hMLH1	Liu and Yang (2015)	2015	China	Plasma	50 IM, 50 Dysplasia, 50 GC, 30 Control	MSP	IM: 20.0, Dysplasia: 44.0, GC: 48.0	—	96.7	—
PCDH10	Pimson et al. (2016)	2016	Thailand	0.2 mL plasma	101 GC, 202 Control	MSP	94.1	—	97.0	—
BARHL2	Yamamoto et al. (2016)	2016	Japan	Gastric washes	70 GC, 70 Control	Pyrosequencing	—	—	—	—
OSR2	Li et al. (2016)	2016	China	Tissue	48 GC, 25 Control	MSP	70.8	—	96.0	—
				0.4 mL serum	48 GC, 25 Control	MSP	62.5	—	92.0	—
VAV3	Li et al. (2016)	2016	China	Tissue	48 GC, 25 Control	MSP	54.2	—	100.0	—
				0.4 mL serum	48 GC, 25 Control	MSP	45.8	—	100.0	—
PPFIA3	Li et al. (2016)	2016	China	Tissue	48 GC, 25 Control	MSP	60.4	—	96.0	—
				0.4 mL serum	48 GC, 25 Control	MSP	56.3	—	96.0	—
HOXD10	Lin et al. (2017)	2017	China	0.4 mL plasma	131 GC, 56 IN, 30 IM, 34 Control	MSP	GC: 48.1	—	80.0	—
FLT3	Maeda et al. (2018)	2018	Japan	Tissue	52 GC, 50 Control	Pyrosequencing	—	—	—	0.77
LINC00643	Maeda et al. (2018)	2018	Japan	Tissue	52 GC, 50 Control	Pyrosequencing	—	—	—	0.76
JAM2	Maeda et al. (2018)	2018	Japan	Tissue	52 GC, 50 Control	Pyrosequencing	—	—	—	0.73
BHLHE22	Maeda et al. (2018)	2018	Japan	Tissue	52 GC, 50 Control	Pyrosequencing	—	—	—	0.72
RIMS1	Maeda et al. (2018)	2018	Japan	Tissue	52 GC, 50 Control	Pyrosequencing	—	—	—	0.76
GUSBP5	Maeda et al. (2018)	2018	Japan	Tissue	52 GC, 50 Control	Pyrosequencing	—	—	—	0.70
ZNF3	Maeda et al. (2018)	2018	Japan	Tissue	52 GC, 50 Control	Pyrosequencing	—	—	—	0.80
PAX5	Haghverdi and Moslemi (2018)	2018	Iran	Blood	35 GC, 35 Control	MSP	28.6	—	100.0	—
SPG20	Wei et al. (2019)	2019	China (Taiwan)	0.5 mL serum	53 GC, 20 control	MSP	88.6	—	75.0	—
RPRML	Alarcón et al. (2020)	2020	Chile	0.5 mL plasma	25 GC, 25 Control	qMSP	56.0	—	88.0	0.73
ZNF582	Peng et al. (2022)	2022	China	3.5 mL plasma	109 GC, 190 Control	qMSP	56.0	—	91.1	0.84
KCNQ5	Li et al. (2022)	2022	China	3.5 mL plasma	Train cohort	qMSP	Train cohort:22.6	Train cohort:12.5	Train cohort: 98.5	Train cohort:0.64
					53 GC, 67 Control					
					Validation cohort1		Validation cohort1: 34.6	Validation cohort1:21.4	Validation cohort1:100.0	Validation cohort1: 0.69
					55 GC, 50 Control					
					Validation cohort2		Validation cohort2:22.8	Validation cohort2: 22.2	Validation cohort2: 100.0	Validation cohort2:0.63
					57 GC, 82 Control					
C9orf50	Li et al. (2022)	2022	China	3.5 mL plasma	Train cohort	qMSP	Train cohort:50.9	Train cohort:37.5	Train cohort: 95.5	Train cohort:0.73
					53 GC, 67 Control					
					Validation cohort1		Validation cohort1:50.9	Validation cohort1:21.4	Validation cohort1:98.0	Validation cohort1:0.74
					55 GC, 50 Control					
					Validation cohort2		Validation cohort2:64.9	Validation cohort2: 44.4	Validation cohort2: 93.9	Validation cohort2:0.82
					57 GC, 82 Control					
CLIP4	Li et al. (2022)	2022	China	3.5 mL plasma	Train cohort	qMSP	Train cohort:37.7	Train cohort:25.0	Train cohort: 92.5	Train cohort: 0.65
					53 GC, 67 Control					
					Validation cohort1		Validation cohort1:25.5	Validation cohort1:21.4	Validation cohort1:92.0	Validation cohort1: 0.60
					55 GC, 50 Control					
					Validation cohort2		Validation cohort2:43.9	Validation cohort2: 33.3	Validation cohort2: 90.2	Validation cohort2: 0.68
					57 GC, 82 Control					

SN, sensitivity; SP, specificity; AUC, area under the curve; AG, atrophic gastritis; GC, gastric cancer; GID, gastrointestinal disease; NED, no evidence of disease; BGD, benign gastric diseases; IN, intraepithelial neoplasia; IM, intestinal metaplasia; GFGP, gastric fundic gland polyp; AP, small adenoma; HP, hyperplastic polyp; GPL, gastric precancerous lesions; MSP, methylation specific PCR; qMSP, quantitative methylation specific PCR; CORD, combined restriction digital PCR; FFPE, Formalin-Fixed and Parrffin-Embedded; MS-MCA, Methylation-sensitive melt curve analysis.

**TABLE 2:** DNA methylation panels for GC detection.

Markers	Authors	Year	Country	Sample types	Number of subjects	Methods	SN (%)	Stage I SN (%)	SP (%)	AUC
P16, E-cadherin and RARb	Koike et al. (2004)	2004	Japan	Serum	41 GC, 10 Control	MSP	44.0	36.8	100.0	—
FAM5C and MYLK	Chen et al. (2012)	2012	China	Serum	58 GC, 46 GPL, 30 Control	MSP	GC: 77.6, GPL: 30.4	—	90.0	0.84
RNF180, DAPK1 and SFRP2	Zhang et al. (2014)	2014	China	0.4 mL plasma	57 GC, 42 Control	MSP	87.7	—	38.1	—
SEPT9 and RNF180	Cao et al. (2020)	2020	China	3.5 mL plasma	74 GC, 57 NED	qMSP	40.5		85.3	0.65
SEPT9, RNF180 and CA7-24	Xu et al. (2021b)	2021	China	3.5 mL plasma	151 GC, 56 AG, 87 Other GIDs, 224 NED	qMSP	68.6	33.3	85.1	—
Reprimo and hMLH1	Liu and Yang (2015)	2015	China	Plasma	50 IM, 50 Dysplasia	MSP	IM: 34.0, Dysplasia: 76.0, GC: 84.0	—	96.7	—
					50 GC, 30 Control					
PYCARD, APAF1, MINT1, and BRCA1	Shin et al. (2016)	2016	Korea	2 mL plasma	41 GC, 104 Control	MSP	97.6	—	66.3	—
OSR2, VAV3, and PPFIA3	Li et al. (2016)	2016	China	0.4 mL serum	48 GC, 25 Control	MSP	83.3	—	88.0	—
ZIC1, HOXD10 and RUNX3	Lin et al. (2017)	2017	China	0.4 mL plasma	131 GC, 56 IN, 30 IM, 34 Control	MSP	91.6	—	50.0	—
ELMO1, ZNF569 and C13orf18	Anderson et al. (2018)	2018	United States	2 mL plasma	36 GC, 38 Control	qMSP	86.0	—	95.0	—
153 cfDNA methylation biomarkers	Ren et al. (2022)	2021	China	Plasma	89 GC, 82 Control	MCTA-Seq	67.0	44.0	92.0	0.87
KCNQ5, C9orf50 and CLIP4	Li et al. (2022)	2022	China	3.5 mL plasma	Train cohort: 53 GC, 67 Control	qMSP	Train cohort:67.9	Train cohort:62.5	Train cohort: 86.6	Train cohort:0.79
					Validation cohort1: 55 GC, 50 Control		Validation cohort1: 65.5	Validation cohort1:42.9	Validation cohort1:90.0	Validation cohort1: 0.81
					Validation cohort2: 57 GC, 82 Control		Validation cohort2:73.7	Validation cohort2: 55.6	Validation cohort2: 84.1	Validation cohort2:0.85
Seven methylation marker panel	Ruan et al. (2023)	2023	China	Plasma	Traing cohort: 54 GC, 79 Control	qMSP	Traing cohort: 80.0	Traing cohort: 80.0	Traing cohort: 65.0	—
					Validation cohort: 117 GC, 309 Control		Validation cohort: 82.0	Validation cohort: 78.0	Validation cohort: 69.0	

SN, sensitivity; SP, specificity; AUC, area under the curve; AG, atrophic gastritis; GC, gastric cancer; GID, gastrointestinal disease; NED, no evidence of disease; BGD, benign gastric diseases; IN, intraepithelial neoplasia; IM, intestinal metaplasia; MSP, methylation specific PCR; qMSP, quantitative methylation specific PCR; MCTA-Seq, methylated CpG tandem amplification and sequencing; cfDNA, cell-free DNA.

**TABLE 3:** DNA Methylation-based pan-cancer test for GC detection.

Cancer types	Markers	Authors	Year	Country	Sample types	Number of subjects	Methods	SN (%)	GC SN (%)	SP (%)	AUC	TOO (%)
GC, CRC	*RASSF2* and *SFRP2*	Nagasaka et al. (2009)	2009	Japan	0.1 g stool	21 GC, 84 CRC, 27 AA, 113 control	Hi-SA	67.6	44.4	89.4	0.78	—
GC, CRC, EC, HCC and LC	cfDNA methylation	Chen et al. (2020)	2020	China	1 mL plasma	Training cohort: 207 healthy, 203 cancers	Targeted bisulfite sequencing	Training cohort: 88.2–91.4	—	Training cohort: 94.7	—	—
						Validation cohort: 207 healthy, 211 cancers		Validation cohort:87.6–94.9		Validation cohort: 96.1		
12 cancer classes (anus, bladder, colon/rectum, esophagus, head and neck, liver/bile duct, lung, lymphoma, ovary, pancreas, plasma cell neoplasm, and stomach)	Targeted methylation	Klein et al. (2021)	2021	United States	10 mL plasma	15,254 participants (56% with cancer, and 44% without cancer), included 30 GC	Bisulfite sequencing	51.5	66.7	99.5	—	88.7
CRC, HCC, ESCC, GC, EAC, PC	cfDNA methylation	Kandimalla et al. (2021)	2021	United States	1–2 mL plasma	46 healthy, 40 CRC, 74 PC, 43 HCC, 12 EAC, 48 ESCC, 37 GC	Targeted bisulfite sequencing	—	—	96.0	0.88	0.53–0.94
GC, EJC and EC	*ELMO1*, *ZNF582* and *TFPI2*	Peng et al. (2022)	2022	China	3.5 mL plasma	109 GC, 29 EJC, 48 EC, 190 Control	qMSP	71.0	67.9	90.0	0.87	—
GC, CRC and EC	Six methylation biomarkers	Ma et al. (2022a)	2022	China	3.5 mL plasma	136 GC, 98 CRC, 48 EC, 195 Control	qMSP	76.6	69.9	89.2	0.90	—
GC and CRC	*SDC2*, *TFPI2*, *WIF1* and *NDRG4*	Ma et al. (2022b)	2022	China	3 g stool	35 GC, 39 CRC, 6 AA, 107 healthy, 30 other cancers	qMSP	68.8	67.5	97.8	—	—
CRC, GC, HCC, EC, and PC	cfDNA methylation and fragmentation signatures	Yang et al. (2023)	2023	China	Plasma	787 healthy, 342 HCC, 239 GC, 209 EC, 180 CRC, and 87 PC	Targeted bisulfite sequencing	86.2	70.3	96.7	0.96	82.0

SN, sensitivity; SP, specificity; AUC, area under the curve; GC, gastric cancer; CRC, colorectal cancer; EC, esophageal cancer; HCC, hepatocellular carcinoma; LC, lung cancer; PC, pancreatic adenocarcinoma; EJC, esophagogastric junction cancer; ESCC, esophageal squamous cell carcinoma; EAC, esophageal adenocarcinoma; AA, advanced adenomas; TOO, tissue of origin; MSP, methylation specific PCR; qMSP, quantitative methylation specific PCR; cfDNA, cell-free DNA; Hi-SA, Fluoroscence High-sensitivity assay for bisulfite DNA.

The methods used for analyzing DNA methylation markers included methylation-specific PCR (MSP), nested MSP, quantitative methylation-specific PCR (qMSP), Sanger sequencing, combined restriction digital PCR (CORD), methylation-sensitive melt curve analysis (MS-MCA), pyrosequencing and bisulfite sequencing (Table 1; Table 2; Table 3). Our analysis revealed that MSP was the most commonly used technology in tissue and plasma/serum samples. However, the reported frequency of MSP has significantly decreased in the past 5 years. Similarly, most reports on pyrosequencing for GC methylation analysis were published 5 years ago and mainly focused on tissue and gastric wash samples. In contrast, qMSP has emerged as a more sensitive and convenient method, and it has been increasingly used for plasma/serum analysis in recent years. While the bisulfite sequencing, especially the target bisulfite sequencing is a commonly used method for pan-cancer early detection in recent years (Table 3).

## 3 DNA methylation markers individually evaluated for GC detection

A total of 59 DNA methylation markers were evaluated individually in 41 studies, and 13 out of the 59 markers (*P16*, *RNF180*, *RUNX3*, *SEPT9*, *E-cadherin*, *Reprimo*, *ELMO1*, *TFPI2*, *RASSF1A*, *SFRP2*, *RPRM*, *ZIC1, and DAPK*) were reported at least 2 times. Among these markers, 37 out of them were analyzed in blood samples (plasma or serum), and the repeatedly evaluated markers were also assessed in blood samples at least once. In addition to blood samples, this review also included markers evaluated in tissue and gastric washes/juice. For instance, *Sox17* (Oishi et al., 2012) and *BARHL2* (Yamamoto et al., 2016) were exclusively evaluated in gastric washes, and 15 markers (*CDO1*, *DCLK1*, *SFRP1*, *ZNF331*, *ZSCAN18*, *FLT3*, *LINC00643*, *JAM2*, *BHLHE22*, *RIMS1*,*GUSBP5*, *ZNF3*, *CHFR*, *NMDAR2B*, and *RECK*) were solely evaluated in tissue samples (fresh frozen tissue or FFPE). The remaining six markers (*MINT25*, *RORA*, *PRDM5*, *MLF1*, *ADAM2*, and *GDNF*) were evaluated simultaneously in both tissue and gastric wash samples (Table 1).

The most frequently evaluated methylated marker for GC early detection was *P16*, which was assessed in 7 studies across various sample types, including tissue, serum, or plasma (Lee et al., 2002; Kanyama et al., 2003; Ichikawa et al., 2004; Koike et al., 2004; Hou et al., 2005; Abbaszadegan et al., 2008; Saliminejad et al., 2020). Nonetheless, the sensitivity of *P16* in blood samples was relatively low in these studies (Lee et al., 2002; Ichikawa et al., 2004; Saliminejad et al., 2020). Apart from *P16*, *RNF180,* and *RUNX3* were the most frequently investigated methylation markers, each mentioned in 5 studies (Table 1). *RNF180* was studied in various research conditions, including marker discovery to multiple center applications (Cheung et al., 2012; Zhang et al., 2014; Cao et al., 2020; Xu J. et al., 2021). Cheung et al. (2012) first reported the feasibility of using *RNF180* methylation as an early detection marker for GC in 0.8 mL plasma, showing a sensitivity of 56.3% and specificity of 100.0%. Three studies using 3.5 mL plasma showed that *RNF180* methylation had sensitivities ranging from 32.4% to 71.7%, with specificities of 59.4%–88.4% (Cao et al., 2020; Xu J. et al., 2021; Zhao et al., 2022a). *RUNX3* also were explored both in tissue and blood samples, its sensitivities in blood range from 29.2% to 58.3%, with the specificities of 79.2%–100.0% (Sakakura et al., 2009; Lin et al., 2017; Hideura et al., 2020; Saliminejad et al., 2020). The three studies examining *SEPT9* analyzed it under identical research conditions, yielding sensitivities of 28.4%–48.3% and specificities of 86.9%–98.2% (Cao et al., 2020; Xu J. et al., 2021; Zhao et al., 2022b). *Reprimo* showed 62.0%–95.3% sensitivities and 90.3%–100.0% specificities in plasma for GC detection (Bernal et al., 2008), and also exhibited 94.3% sensitivity and 92.9% specificity in 1 mL serum (Wang et al., 2016), indicated that it is a promising marker for early detection of GC. *E-cadherin* has exhibited 100.0% specificity in previous studies conducted on three serum cohorts, but its sensitivity remains relatively low, ranging from 22.2% to 57.4% (Lee et al., 2002; Ichikawa et al., 2004; Koike et al., 2004). Pimson et al. (2016) found that methylated *RASSF1A* and *PCDH10* have high sensitivities (83.2% and 94.1%) and specificities (94.6% and 97.0%) in 0.2 mL plasma, while another study demonstrated that *RASSF1A* had only 33.3% sensitivity with a specificity of 100.0% in 2 mL plasma (Saliminejad et al., 2020). The *ZIC1* was evaluated not only in GC samples but also in samples of gastric precancerous lesions, such as intraepithelial neoplasia (IN) and intestinal metaplasia (IM) (Chen et al., 2015; Lin et al., 2017). Other methylation markers such as *SFRP2* (Miao et al., 2020), *RPRM* (Saliminejad et al., 2020), *OSR2* (Li et al., 2016), *PPFIA3* (Li et al., 2016), *ZNF582* (Peng et al., 2022) and *C9orf50* (Li et al., 2022) showed sensitivities higher than 50.0% with specificities higher than 80.0% in plasma, indicating their potential as non-invasive tools for GC early detection.

For early-stage GC detection, most methylation markers lack of the performance evaluation in stage I GC (Table 1). *RNF180* and *SEPT9* both showed sensitivities less than 20.0% in stage I GC (Xu J. et al., 2021). Among the markers evaluated in stage I GC, *C9orf50* had sensitivities ranging from 21.4% to 44.4% (Li et al., 2022), while *P16, E-cadherin* and *SFRP2* showed the relatively higher sensitivities ≥50.0% (Lee et al., 2002; Miao et al., 2020). Only one study containing methylated *KCNQ5*, *C9orf50*, and *CLIP4* was evaluated in multiple cohorts and showed good reproducibility in three plasma cohorts (Li et al., 2022).

## 4 DNA methylation panels for GC detection

Single DNA methylation markers for GC detection often exhibit insufficient sensitivity, particularly for early-stage cancer, due to tumor heterogeneity and individual differences. Developing methylation panels using multiple DNA methylation markers is an effective strategy to improve sensitivity and has been successfully applied in detection of CRC (Zhao et al., 2019; Zhao et al., 2020) and lung cancer (LC) (Zhang et al., 2017; Wei et al., 2021). In the past decade, several methylation markers were evaluated in panels for GC early detection, with all studies using blood samples, including 10 plasma and three serum sample cohorts (Table 2). The *SEPT9* and *RNF180* combination was the first and only non-invasive panel approved by NMPA for plasma GC detection in 2020. A previous study indicated that the combination of *SEPT9* and *RNF180* improved sensitivities from 28.4%–32.4%–40.5% with a specificity of 85.3% (Cao et al., 2020). Another study using a combination of *SEPT9*, *RNF180*, and CA72-4, achieved 33.3% and 68.6% sensitivities for stage I and overall stage GC, with a specificity of 85.1% (Xu J. et al., 2021). Combining *RNF180* with other markers, such as *DAPK1* and *SFRP2*, showed a high sensitivity of 87.7% but a lower specificity of 38.1% (Zhang et al., 2014).

The combination of *P16*, *E-cadherin*, and *RARb* in a panel resulted in a significant improvement in sensitivity compared to using a single marker. However, the sensitivity of the panel remained relatively low (Koike et al., 2004). In contrast, the combination of *FAM5C* and *MYLK* demonstrated an area under the curve (AUC) of 0.84 for GC detection, indicating its potential as a non-invasive blood-based method for early detection of GC (Chen et al., 2012). One panel including *Reprimo* and *hMLH1* identified 84.0% of GC cases with a specificity of 96.7%. This panel could also be expanded to detect gastric dysplasia and IM with sensitivities of 76.0% and 34.0%, respectively (Liu and Yang, 2015). Shin et al. (2016) reported a methylation panel involving four new markers, *PYCARD*, *APAF1*, *MINT1*, and *BRCA1*, with a sensitivity and specificity of 97.6% and 66.3%. Two other studies used low volume blood samples and included three methylation markers by using MSP, showing an increasing trend of sensitivity but a decline in specificity when compared to single markers (Li et al., 2016; Lin et al., 2017). Anderson et al. (2018) developed and validated a novel panel including methylated *ELMO1*, *C13orf18,* and *ZNF569*, which demonstrated a promising sensitivity of 86.0% and a specificity of 95.0%. Ren et al. (2022) used methylated CpG tandem amplification and sequencing (MCTA-Seq) to develop a panel with 153 cfDNA methylation markers, which could detect 44.0% of stage I GC in plasma. Li et al. (2022) integrated *KCNQ5*, *C9orf50* and *CLIP4* in a single tube qMSP panel, and evaluated its performance in three cohorts, finding that it had about 10.0%–30.0% in increase of sensitivity compared with single markers. Ruan et al. (2023) also developed a qMSP panel included seven methylation markers, and validated in two independent cohorts, achieving sensitivities and specificities of 80.0%–82.0% and 65.0%–69.0%, respectively.

## 5 DNA methylation-based pan-cancer test for GC detection

Except for methylation panels for single cancer type early detection, detecting multiple cancer types together in one panel, called a pan-cancer test, is a new strategy for reducing cancer morbidity and mortality (Duffy et al., 2021; Jamshidi et al., 2022). Seven pan-cancer tests based on methylation markers have been summarized in this review, which have been applied to at least two cancer types, including GC (Table 3). Four of these tests were developed using bisulfite sequencing, all of which demonstrated high specificities higher than 95.0% (Chen et al., 2020; Kandimalla et al., 2021; Klein et al., 2021; Yang et al., 2023). Moreover, most of the bisulfite sequencing based pan-cancer tests can identify tissue of origin (TOO) (Table 3). As for qMSP-based method, Peng et al. (2022) developed a panel by combined using *ZNF582*, *ELMO1,* and *TFPI2*, which can detect GC, esophageal cancer (EC) and esophagogastric junction cancer (EJC) together, and achieved 67.9% and 71.0% sensitivities for GC and all cancer types, respectively, with a specificity of 90.0%. Ma Y. et al. (2022) reported a six methylation markers panel for detection of EC, GC and CRC in plasma, it showed 69.9% and 76.6% for GC and all cancer types with a specificity of 89.2%. In addition to blood, stool was also utilized as a sample type for detection of the pan-cancer in gastrointestinal tract. For example, methylation *RASSF2* and *SFRP2* were combined using for detection of CRC and GC in 0.1 g stool samples (Nagasaka et al., 2009), and *Ma* et al used 3 g stool as a sampling type for simultaneous detection of CRC and GC, and it could detect 67.5% GC with a specificity of 97.8% (Ma L. et al., 2022).

## 6 Challenges of GC early detection using DNA methylation

**FIGURE 1:**
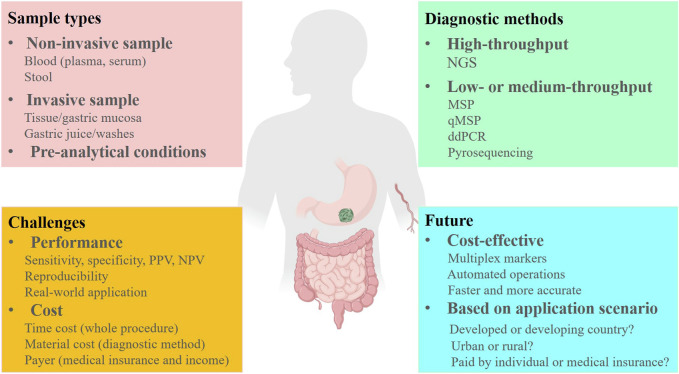
The challenges and future of DNA methylation markers for early detection of GC. PPV, positive predictive value; NPV, negative predictive value. Created with MedPeer (www.medpeer.cn).

Indeed, DNA methylation-based cancer early detection tests face several challenges that need to be addressed before they can be widely adopted in clinical settings (Figure 1). One of the major challenges is the complexity of the DNA methylation detection process. The process often involves bisulfite treatment, PCR amplification, and sequencing or other detection methods. These steps can introduce errors or biases into the results, which can affect the accuracy and reproducibility of the test. In addition to the technical challenges, there are several other factors that can impact the performance of DNA methylation-based cancer early detection tests. For example, the quality of the sample is critical, and the performance of the test can be affected by the pre-analytical conditions, such as the time and temperature of sample storage and transportation. Moreover, the accuracy of the test can be influenced by the selection of the target CpG sites and the panel design. It is important to ensure that the selected CpG sites are informative and specific for the target cancer types, and that the panel design is optimized for sensitivity and specificity. Another critical factor is the quality of the enrolled subjects and the selection criteria. DNA methylation-based cancer early detection tests may have limited sensitivity in early-stage cancers, and false positive results may occur in some cases. Therefore, it is important to carefully select the enrolled subjects based on their clinical and pathological characteristics, such as age, gender, tumor stage, and histological type, to minimize the risk of false positives or false negatives. Lastly, the quality of the operators and the analytical methods used for the test are also important factors that can affect the performance and reproducibility of the test. Therefore, it is crucial to establish standard operating procedures and quality control measures to ensure that the test results are accurate and reliable.

As mentioned in this review, the samples involved in GC DNA methylation tests include fresh frozen tissue, FFPE, plasma, serum, gastric washes/juice, and stool (Figure 1). However, fresh frozen tissue, FFPE, and gastric washes/juice are invasive sample types and therefore not suitable for large-scale screening or early diagnosis of GC. Tissue samples are more appropriate for discovering DNA methylation markers rather than early detection. In comparison, the methylation level of each marker is consistently higher in tissues than in plasma and serum, which due to the proportion of circulating tumor DNA (ctDNA) in the blood is much lower than that of tumor DNA in the tissues (Abbosh et al., 2018). Therefore, when we translate the markers that from discovery stage in GC tissues to blood-based assay development, a significant decrease in sensitivities might be observed in blood samples (Table 1).

Blood is a convenient, non-invasive, and high-throughput processable sample, that is, easily accessible. Plasma and serum are two main sample types for blood ctDNA analysis, as indicated by the studies summarized in this review (Table 1). Previous head-to-head studies have shown that plasma is a preferable sample type for ctDNA analysis because the ctDNA fraction in serum is lower than that in plasma, while the background and large DNA fragments are higher in serum (Lee et al., 2020; Pittella-Silva et al., 2020). Therefore, we suggest plasma as the ideal non-invasive sample type for GC early detection, and recent studies on GC early diagnosis have also confirmed that plasma is a superior sample type (Table 1; Table 2; Table 3). However, an important issue to address is the significant variation in the volume of plasma samples used in different studies (Table 1), which can have a significant impact on the performance of DNA methylation tests and affect subsequent marker selection and replication by other researchers. Furthermore, the standardized pre-analytical procedure must be considered before the clinical applications (Figure 1) (Kerachian et al., 2021). For example, blood drawn by EDTA tubes should be processed within 4–6 h after collection if storage at room temperature (Meddeb et al., 2019), and stored at 4°C in EDTA tubes for up to 24 h (Kerachian et al., 2021). During the plasma fraction separation procedure, the brake function of the centrifuge must be turned off to prevent disruption of the cell layer (Kerachian et al., 2021), and centrifuging the blood twice is recommended (Volckmar et al., 2018). The plasma should be processed for cfDNA isolation within 24 h if stored at 4°C, and for long-term storage at −20 or −80°C (Kerachian et al., 2021).

Stool is an easily accessible and non-invasive sample type that can be conveniently collected at home (Qian, 2017), making it an ideal sample for early detection of gastrointestinal cancers. However, DNA derived from the stomach has a longer residence time in the digestive tract, making it more susceptible to degradation by nucleases and gastric acid present in the gastrointestinal tract (Olson et al., 2005; Liu et al., 2015). As a result, the proportion of DNA originating from the stomach in stool samples is relatively lower compared to fresh DNA derived from the colon. Consequently, the use of stool samples for detecting GC often exhibits significantly lower sensitivity compared to the detection of CRC (Table 3).

The most commonly used analytical method for analyzing DNA methylation is the bisulfite-treat-based method, which includes MSP, qMSP, bisulfite Sanger sequencing, bisulfite next-generation sequencing (NGS), and pyrosequencing (Figure 1) (Kurdyukov and Bullock, 2016). The entire process consists of three steps: 1) DNA isolation from specimens; 2) DNA bisulfite treatment and purification; 3) converted DNA analysis. The efficiencies of the DNA isolation kit and the DNA bisulfite treatment kit are crucial factors that affect the performance of DNA methylation analysis (Cox et al., 2022). Some studies have compared the most commonly used commercial cfDNA isolation kits and bisulfite conversion kits and have observed significant differences in cfDNA recovery efficiency and bisulfite conversion efficiency (Sorber et al., 2017; Worm Ørntoft et al., 2017). Hence, carefully selecting a suitable and highly efficient kit is necessary during DNA methylation assay development and application. Among the DNA methylation analytical methods, MSP is an economical and traditional method that has been widely used in various sample types (Ramalho-Carvalho et al., 2018), but its low resolution, low-throughput, and potential for cross-contamination limit its application in liquid biopsy (Mao and Chou, 2010). qMSP, a modified method combining MSP and qPCR, can detect several DNA methylation markers simultaneously in one tube with high resolution and avoid cross-contamination from gel analysis (Sigalotti et al., 2019). Currently, several qMSP-based non-invasive cancer early detection tests have approved by FDA and NMPA due to their cost-effectiveness and convenience (Imperiale et al., 2014; Potter et al., 2014; Wu et al., 2016; Wang et al., 2020). Bisulfite NGS as a high-throughput analytical method was used for DNA methylation markers discovery and large-panel development (Luo et al., 2020), and its wider coverage of DNA markers can avoid false positives. However, the data summarized in this review suggest that the NGS-based GC early detection panels do not offer a significant advantage compared to qMSP-based panels (Table 2; Table 3), and the high-cost and complex operation process also limit their application.

In the field of GC early detection, the current methylation markers being used are predominantly pan-cancer markers rather than GC-specific markers. For example, *SEPT9* is approved by the FDA and NMPA as a plasma marker for CRC detection (Potter et al., 2014; Wu et al., 2016), while *SFRP2* (Li et al., 2019), *KCNQ5* (Jensen et al., 2019), *C9orf50* (Jensen et al., 2019), *CLIP4* (Jensen et al., 2019) and *TFPI2* (Hibi et al., 2011b) were found to be positive in CRC plasma. Similarly, *ZNF582* (Huang et al., 2017) and *ZNF569* (Salta et al., 2020) showed high methylation in esophageal cancers, and *RASSF1A* has been approved by NMPA as a lung cancer early detection marker (Wei et al., 2021). Meanwhile, the above data in GC samples all came from case-control study, and have not been evaluated in the real-world asymptomatic population. While the cfDNA in blood is derived from organs throughout the body, thus the blood-based GC early detection may result in numerous “false positives” in real-world application if using the pan-cancer methylation markers. To address the issue of insufficient specificity for current methods, a pan-cancer panel may be utilized, but it also raises concerns regarding the next-step examination in case of a positive result. On the bright side, many methylation markers are more likely to exhibit positivity in the digestive system while being relatively specific in other organs. Therefore, the optimal solution for GC early detection in the future may be the target digestive system pan-cancer test.

## 7 Future of GC early detection using DNA methylation

**FIGURE 2:**
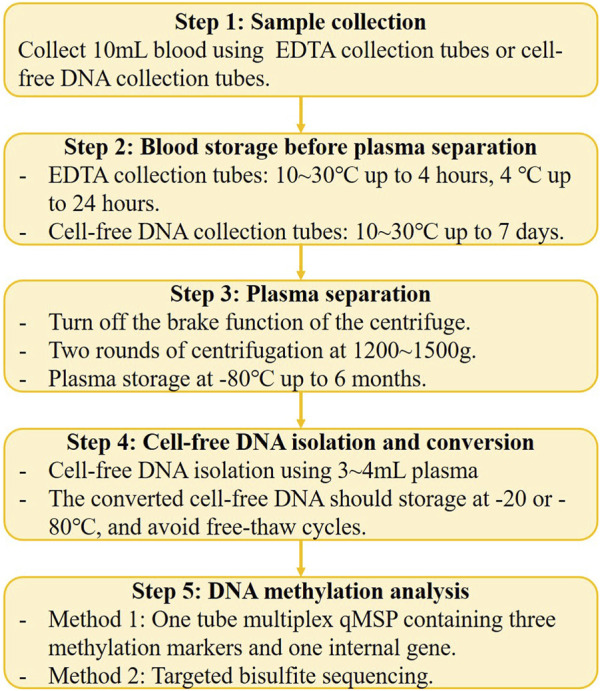
The potential standardized flowchart for plasma DNA methylation analysis.

Compared to DNA methylation in CRC early detection (Nikolaou et al., 2018; Worm Ørntoft, 2018; Nassar et al., 2021), the recent milestones of methylation-based GC early detection are still in a relatively early stage, and the majority of the GC methylation studies were focused on the Asian region, especially in China and Japan (Table 1). In the future, the researchers should pay more attention to the discovery of specific markers and improve the early-stage GC sensitivity, such as through enrichment of the short-length ctDNA in plasma (Mouliere et al., 2018). Meanwhile, the cost and the payer condition (individual or medical insurance) are two important factors that must be concerned when developing and applying the GC DNA methylation test (Figure 1). In developing countries and rural regions, a qMSP-based test may be the first choice, but in developed countries and urban areas, NGS-based methods can also be an alternative solution. Furthermore, automating pre-analytical procedures will provide more consistent and reproducible detection results and reduce costs in the future (Figure 1). Finally, multiplex DNA methylation detection based on blood sample might the optimal method for early detection of GC in the future, which can avoid individual biases by combining multiple markers and improve sensitivity for early-stage cancer detection. Therefore, we provide a potential standardized flowchart for plasma DNA methylation analysis in Figure 2 based on our experience and literature.

## 8 Conclusion

In conclusion, DNA methylation as a robust and sensitive marker has also been widely studied in GC samples, numerous potential DNA methylation markers have already been identified in GC, and some of them have been developed as commercial kits. But the lack of early-stage GC sensitivity and specificity should be improved in the future, and the standardized sampling, pre-analytical, cfDNA isolation and conversion procedures must be considered in development of the assay. With the rapid development of new technology and the discovery of more methylation markers, it is expected that DNA methylation will become a cost-effective and non-invasive tool for GC early detection in the near future.

## Figures and Tables

**Table:** 

**SN**	Sensitivity
**SP**	Specificity
**AUC**	Area Under the Curve
**AG**	Atrophic Gastritis
**GC**	Gastric Cancer
**CRC**	Colorectal Cancer
**EC**	Esophageal Cancer
**HCC**	Hepatocellular Carcinoma
**LC**	Lung Cancer
**PC**	Pancreatic Adenocarcinoma
**EJC**	Esophagogastric Junction Cancer
**ESCC**	Esophageal Squamous Cell Carcinoma
**EAC**	Esophageal Adenocarcinoma
**AA**	Advanced Adenomas
**GID**	gastrointestinal Disease
**NED**	No Evidence of Disease
**BGD**	Bbenign Gastric Diseases
**IN**	Intraepithelial Neoplasia
**IM**	Intestinal Metaplasia
**GFGP**	Gastric Fundic Gland Polyp
**AP**	Small Adenoma
**HP**	Hyperplastic Polyp
**MSP**	Methylation Specific PCR
**qMSP**	quantitative Methylation Specific PCR
**CORD**	Combined Restriction Digital PCR
**FFPE**	Formalin-Fixed and Parrffin-Embedded
**ctDNA**	circulating tumor DNA
**cfDNA**	cell-free DNA
**MCTA-Seq**	Methylated CpG Tandem Amplification and Sequencing
**TOO**	Tissue of Origin
**PPV**	Positive Predictive Value
**NPV**	Negative Predictive Value
**FDA**	Food and Drug Administration
**NMPA**	Chinese National Medical Products Administration
